# Virus interactions with bacteria: Partners in the infectious dance

**DOI:** 10.1371/journal.ppat.1008234

**Published:** 2020-02-11

**Authors:** Ursula Neu, Bernardo A. Mainou

**Affiliations:** 1 Institute of Chemistry and Biochemistry, Freie Universität Berlin, Berlin, Germany; 2 Department of Pediatrics, Emory University School of Medicine, Atlanta, Georgia, United States of America; 3 Children’s Healthcare of Atlanta, Atlanta, Georgia, United States of America; Mount Sinai School of Medicine, UNITED STATES

The outcome of viral infection depends on the interplay between host factors and the environment. Host factors, like the expression of viral receptors, convey permissiveness to infection, define tropism, regulate antiviral immune responses, determine viral clearance, and spread. The host microbiota, the constellation of microbes inhabiting an organism, also plays a key role in the outcome of infection. Microbes and microbial products can directly interact with viral particles. Our understanding of how the microbiota impacts virus infection is largely limited to the bacterial component of the microbiota. Although bacteria do not support eukaryotic virus infection, they can promote viral fitness by enhancing virion stability, promoting infection of eukaryotic cells, and increasing coinfection rates. Virus binding of bacteria can also impact bacterial biology, including bacterial adherence to eukaryotic cells. These interactions can also indirectly affect the host response to viral infection. In this Pearl, we focus on how direct and indirect interactions between viruses and bacteria impact viral biology and touch on recent findings that illustrate how bacterial biology can also be impacted by interactions with eukaryotic viruses (Fig 1).

**Fig 1:**
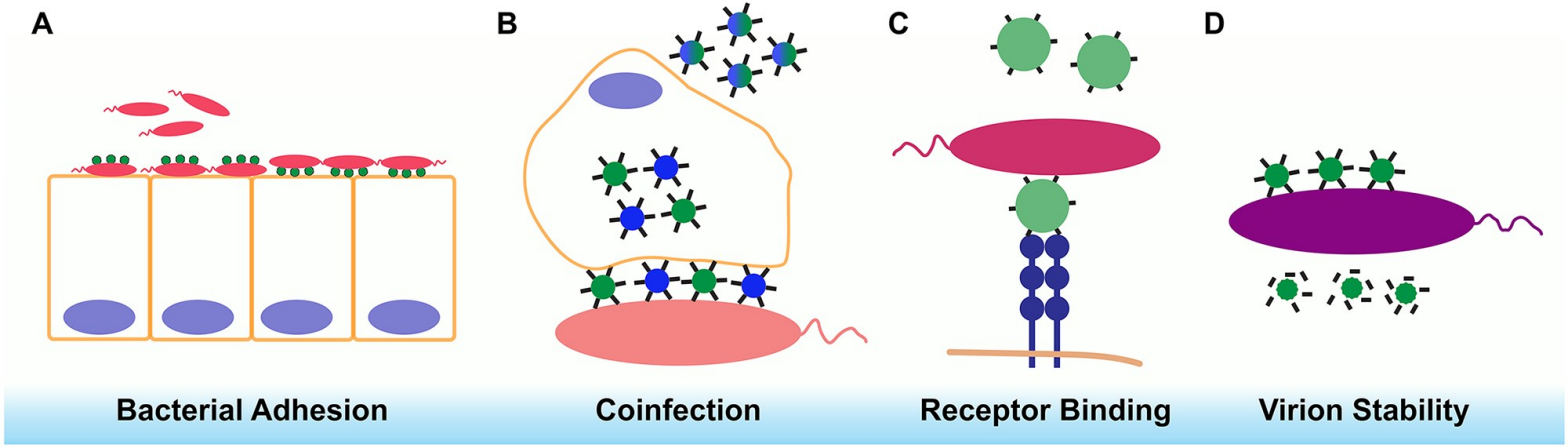
Virus interactions with the microbiota impact various aspects of microbial biology. (A) Binding of influenza A virus to bacteria enhances bacterial adhesion to eukaryotic cells. (B) Binding of multiple poliovirus virions to bacteria results in enhanced coinfection and genetic recombination, giving rise to reassortant viruses. (C) Poliovirus binding to LPS enhances its affinity for PVR and binding of human norovirus to bacterial histo-blood group antigens promotes infection. (D) Binding of gram-positive and gram-negative bacteria by picornaviruses and mammalian reovirus enhances virion thermostability. LPS, lipopolysaccharide; PVR, poliovirus receptor.

## Direct interactions between mammalian viruses and the microbiota

The bacterial component of the microbiota can directly or indirectly impact the outcome of infection by a range of different viruses. Direct interactions have been observed between bacteria and influenza A virus (IAV) [1, 2] as well as several enteric viruses: picornaviruses (including poliovirus [3, 4]); coxsackieviruses A21, B2, B3, Echovirus 30, Mengo, and Aichi viruses [5, 6]; human noroviruses (HNoV) [7, 8]; and mammalian orthoreovirus (reovirus) [9]. Although bacteria can directly impact the outcome of infection by several viruses, the viral factors involved in the interaction between bacteria and viruses are largely undefined.

In many cases, binding of viruses to bacteria is mediated through bacterial envelope components lipopolysaccharide (LPS), the main component of the gram-negative bacterial envelope, and peptidoglycan (PG), the main component of the gram-positive bacterial envelope. Poliovirus binds to LPS and PG from several bacterial species [3–5, 10]. Although the bacterial binding epitopes for poliovirus are unknown, the virus may bind LPS, PG, and chitin through the monosaccharide N-acetyl-glucosamine (GlcNAc) [4]. HNoVs use histo-blood group antigens (HBGAs) to attach to eukaryotic cells [11] and can bind bacterial HBGAs [12]. Reovirus thermostability is enhanced by LPS and PG independent of serotype, but lipoteichoic acid, a major component of the gram-positive bacterial envelope, elevates the thermostability of only one reovirus serotype [9]. As different viral strains and serotypes differ in their interactions with bacterial envelope components, specific genetic determinants of norovirus, poliovirus, and reovirus, likely determine the use of specific bacterial components.

## Molecular and structural determinants of interactions between bacteria and viruses

Bacterial molecules like LPS and PG are large carbohydrate polymers, whereas HBGAs are short carbohydrate motifs. Information on virus–carbohydrate interactions in the context of eukaryotic cells [13] may inform how viruses engage bacterial cells. In general, carbohydrate binding sites on viruses are shallow, water-exposed grooves on the virion surface, leading to weak affinity of single binding sites [14–16]. Viruses use multivalent interactions engaging several binding sites to recognize multiple receptor molecules on host cells. Small sequence variations in viral carbohydrate binding sites can result in big effects on viral tropism and spread [2, 17]. As LPS and PG consist of multiple repeats of smaller subunits, it is possible that viruses engage these molecules at multiple binding sites. Although carbohydrates play an important role in the binding of bacteria by eukaryotic viruses, the bacterial envelope contains other molecules beyond carbohydrates, including proteins [18]. As such, it is possible that other, as-yet-unidentified molecules are involved in the interaction between viruses and bacteria.

The viral proteins that interact with bacterial surfaces have different structures and folds. A residue in an exposed loop of the VP1 capsid protein of poliovirus influences LPS binding [4]. It is not known if the same binding site is used by poliovirus to bind LPS and PG. Reovirus virions and cell entry intermediates (infectious subvirion particles [ISVPs]) are stabilized by LPS and PG, suggesting the virus binds LPS and PG through the viral attachment fiber σ1 [9]. In contrast to poliovirus VP1, which intimately interacts with other capsid proteins [19], reovirus σ1 is a fibrous protein that protrudes up to 40 nm from the virion surface [20]. At least in the context of poliovirus VP1 and reovirus σ1, there is not a shared structure or fold that could be used to predict bacterial envelope component binding.

## Bacteria and bacterial components influence virion stability

Virion stability is tightly controlled. The virion needs to be stable enough to protect the viral genome from environmental exposure during transmission but malleable enough to allow disassembly and viral genome release during cell entry. Enteric viruses use components of the bacterial envelope to enhance virion stability. Direct binding to gram-positive and gram-negative bacteria enhances the thermostability of poliovirus [10], Coxsackievirus B3 [5], HNoV [7, 8], and reovirus [9], whereas the thermostability of Mengo and Aichi picornaviruses is strengthened by gram-positive and gram-negative bacteria, respectively [5]. Interestingly, both gram-positive and gram-negative bacteria also provide protection from bleach treatment to Aichi, Mengo, and poliovirus [5]. Moreover, binding of HNoV to HBGAs protects the virus from heat stress [8].

The stabilizing effects of bacteria extend to viral interactions with host cells. Bacteria enhance poliovirus attachment to host cells [10], and LPS strengthens attachment of poliovirus to poliovirus receptor (PVR) in a dose-dependent manner [4]. Picornaviruses undergo breathing motions, reversible and concerted conformational changes of the capsid at physiological temperatures [21]. PVR binding catalyzes capsid expansion by similar motions during uncoating [22–24]. It is possible that LPS binding increases PVR binding by influencing the conformational equilibrium of the capsid. In the case of reovirus, the attachment fiber σ1 undergoes conformational changes following binding to sialic acid [25]. Although bacteria or bacterial components do not impact reovirus attachment to cells [9], the interaction with bacteria or envelope components may promote a more thermostable σ1 conformation.

Bacteria and their products can also detrimentally impact virion stability and infectivity. Segmented filamentous bacteria protect mice from rotavirus infection independent of interferon, IL-17, and IL-22 [26]. Fecal transfer from mice bearing segmented filamentous bacteria also protects susceptible animals from infection with IAV, vesicular stomatitis virus, and reovirus. Bacterial products can also impact viral infectivity. Surfactin, a cyclic lipopeptide with membrane disruptive properties produced by *Bacillus subtilis* [27], disrupts coronavirus virion integrity and impairs the infectivity of several enveloped viruses, including Chikungunya, Crimean–Congo hemorrhagic fever, Dugbe, Ebola, IAV, Mayaro, Nipah, Una, and Zika [28]. It is conceivable that as-yet-unidentified metabolites and natural products produced by the bacterial component of the microbiota impact viral infectivity.

## Microbial effects on coinfection and tropism

The binding of poliovirus to bacteria enhances coinfection by promoting the delivery of multiple virions to a single cell [3]. Coinfection results in enhanced rates of recombination, which can increase fitness of the viral progeny. These data indicate that poliovirus not only gains higher thermostability during transmission from its interaction with bacteria but also raises its effective multiplicity of infection by more efficiently binding PVR and increasing the rates of superinfection. The resulting rates of genetic recombination from superinfected cells results in enhanced viral fitness.

Infectivity and tropism of HNoV and murine norovirus (MNoV) are impacted by bacteria and bacterial products [12, 29]. Whereas Ruminococcaceae and *Faecalibacterium* spp. negatively affect HNoV infection through the modulation of virus-specific antibody titers [30], commensal bacteria that produce HBGAs promote infection of B cells [12]. NoV infection in the gut is modulated by bile acids, which are modified by the intestinal microbiota to secondary bile acids [31]. Bile acids directly bind HNoV [32] and enhance MNoV cell attachment by promoting engagement of its receptor CD300lf [33] through the regulation of capsid conformational changes [34]. CD300lf is expressed on tuft cells in the gut, and the presence of enteric bacteria, IL-4, or IL-25 regulate the number of tuft cells in the gut [35]. As such, cytokines produced in response to microbes in the gut can impact MNoV infection by affecting the number of cells that are susceptible to infection. It is also clear that caliciviruses have evolved distinct mechanisms to utilize bacteria, bacterial components, or bacterial-modified components to enhance infectivity.

The interaction of viruses with bacteria can also impact bacterial biology. IAV directly binds gram-positive *Streptococcus pneumoniae* and *Staphylococcus aureus*, as well as gram-negative *Moraxella catarrhalis* and *Haemophilus influenzae* [1, 2]. These interactions lead to enhanced bacterial adherence to epithelial cells and increased uptake by macrophages [1, 2]. The interaction of IAV with bacteria also enhances the translocation of bacteria into the middle ear and results in higher mortality in mice than either agent alone [2]. These data provide a mechanistic understanding of clinical observations showing synergistic morbidity and mortality during *S*. *pneumoniae* and IAV coinfections [36]. Similar to IAV, respiratory syncytial virus directly binds *S*. *pneumoniae* via penicillin-binding protein 1a on the bacterial cell and this binding results in increased bacterial adherence to epithelial cells in vitro and in a small animal model [37, 38]. These data suggest that the interaction of viruses with bacteria can potentially benefit both microbes and is likely to extend to other sites where viruses and bacteria interact.

## Modulation of innate and adaptive immune responses by the microbiota

Commensal bacteria are essential for the development of a mature innate and adaptive immune system [39, 40]. Not surprisingly, viruses can use the microbiota and microbial components to modulate the innate immune response to infection. Mouse mammary tumor virus (MMTV) incorporates LPS-binding molecules, including the innate immune Toll-like receptor 4 (TLR-4), into its envelope to bind bacterial LPS [41]. MMTV-bound LPS stimulates TLR-4 signaling in the host and creates an IL-10-dependent immunosuppressive environment that allows viral persistence [42]. Another key component of the innate immune response, Type III interferon (IFN), plays a crucial role in regulating MNoV infection. Enteric bacteria counteract the Type III IFN response, enabling the establishment of persistent infection [29]. Interestingly, the induction of Type III IFN by murine astrovirus can provide protection against MNoV [43], indicating that the interplay between viruses and microbiota extends beyond bacteria.

Commensal bacteria can influence the production of secretory immunoglobulins (sIG), which are secreted into the intestinal lumen and act as the first line of mucosal defense against enteric pathogens [44]. Surprisingly, sIGs promote acute MNoV and reovirus infection through the regulation of IFNγ and inducible nitric oxide synthase (iNOS) levels in the gut [45]. Also, the antibody response to rotavirus infection is impaired by the presence of enteric bacteria [46] and the presence of bacteria can influence vaccine efficacy. Coadministration of inactivated IAV and pneumococcal vaccines enhances pneumococcal- and IAV-specific immune responses in the lung [1, 47]. The mechanism that underlies the enhanced response to pneumococci and IAV is not completely clear, although it is at least in part due to increased viral uptake by antigen-presenting cells.

Over the last decade, our understanding of the various ways that the bacterial component of the microbiota impact viral biology has greatly expanded. Despite these efforts, we still lack a mechanistic understanding of how bacteria and bacterial components influence viral stability, infectivity, and pathogenesis. Although there is overlap in themes of how viruses use bacteria to their advantage, future studies are likely to identify mechanistic differences between viruses that may help explain the varied outcomes of infection observed between viruses.
